# The Effects of a New Public Medicine Procurement Policy on Medicine Price in Shaanxi Province, Western China: An Interrupted Time Series Analysis

**DOI:** 10.3389/fphar.2019.00950

**Published:** 2019-08-29

**Authors:** Shuchen Hu, Chen Chen, Shengfang Yuan, Fei Xue, Li Shi, Yu Fang, Caijun Yang

**Affiliations:** ^1^Department of Pharmacy Administration and Clinical Pharmacy, School of Pharmacy, Xi’an Jiaotong University, Xi’an, China; ^2^Center for Drug Safety and Policy Research, Xi’an Jiaotong University, Xi’an, China

**Keywords:** medicine price, interrupted time series analysis, medicine procurement policy, policy effect, China

## Abstract

**Objectives:** To assess the effects on medicine price, a new public medicine procurement policy (NPMPP) undertaken in western China in 2015.

**Methods:** An interrupted time series analysis was used to evaluate the impact of NPMPP on the prices of emergency medicines, gynaecological medicines, and paediatric medicines in Shaanxi Province, western China. Based on the procurement records in all the public health institutions in Shaanxi Province, we built three regression models. The monthly average price growth rate of the three categories of medicines was analysed covering the period 2015 to 2017.

**Findings:** Before the intervention, there was an increasing trend in the monthly average growth rate of the three categories of medicines, but significant only in emergency medicines and paediatric medicines. After the introduction of NPMPP, the increasing trend was accelerated for both the emergency medicines (coefficient = 0.114, *P* < 0.001) and gynaecological medicines (coefficient = 0.078, *P* < 0.05), whereas the increasing trend for paediatric medicines was slowed down after the intervention (coefficient = −0.024, *P* < 0.05).

**Conclusion:** Using interrupted time series analysis, we identified a statistically significant increase in the price growth rate of emergency medicines and gynaecological medicines, but a statistically significant decrease in the price growth rate of paediatrics, following the introduction of NPMPP. The impact of NPMPP on emergency medicines was greater than that on gynaecological medicines. To inhibit the growth trend of drug price, effective policies need to be introduced.

## Introduction

Drug shortages were a serious problem in China (Fan et al., 2018). By literature review, 148 drugs were identified in shortage from 2006 to 2015, and most of which were cheap and essential medicines (Wu et al., 2016). During the period of 2016 to 2017, the number of drugs in short supply monitored by the central government reached 1,000 (Wang et al., 2018). In a 29-province study in China, nearly two-thirds of the physicians reported that drug shortages occurred at least once a month (Yang et al., 2018). Among the three geographical regions (i.e., eastern, central, and western China), the eastern and western regions experienced more serious drug shortages than did the central region (Fan et al., 2018). One study in Shaanxi Province, western China, reported that there were almost 100 drugs in short supply in only 20 hospitals (12 secondary hospitals and 8 tertiary hospitals) in 2016 (Yang et al., 2016). The ongoing drug shortages crisis had exacted a significant effect on patient care and brought great challenges for the government (Du et al., 2018).

To address this drug shortage problem, the Chinese government employed several pharmaceutical policies. A new public medicine procurement policy (NPMPP) was introduced in January 2015, as the old procurement scheme was criticised as the primary cause of drug shortage (Cui and Lyu, 2018; Xiong et al., 2019). Previously, each province had a medicine tender bidding centre to select medicine manufacturers and to determine medicine prices for government-run healthcare institutions using a “2-envelope” system (Hu, 2017). The first envelope was quality, and the second envelope was the price (Hu, 2017). A composite score was calculated to determine the winner (usually the lowest price wins). To win the tender, the manufacturers usually offered a very low price that would not change for a long time (typically 5 years for most provinces) (Hu, 2017). In the NPMPP, as long as the manufactures met the technical and quality requirements, these manufacturers could be the procurement sources for all the public hospitals (NHFPC, 2015). Besides, manufacturers could negotiate on medicine price with healthcare institutions more frequently (NHFPC, 2015). Therefore, the situation of drug shortage caused by fixed low price may be greatly mitigated under the new medicine procurement scheme (Fang et al., 2015).

The central government issued the NPMPP at first, and then provincial governments started to implement it successively (SNHFPC, 2015). Up to now, the NPMPP has been implemented in 23 provinces. Unlike other types of medicines, there was no ceiling price when the NPMPP was implemented in paediatric, gynaecological, and emergency medicines (used in the emergency department and the prehospital setting).

After the implementation of NPMPP, the shortages of some low-priced medicines have been eased to a certain degree. However, at the same time, the prices of some medicines were reported to rise substantially since the NPMPP (Baidu Research, 2019; Financial Network, 2019; Quanzhou Network, 2019). One study carried out in a hospital in Guangxi Province found that, after the implementation of the NPMPP, the average price increased by 53% in 2016 and 93% in 2017 for 43 medicines (Zheng, 2018). Compared with other kinds of medicines, paediatric, gynaecological, and emergency medicines usually have lower market demands or fewer manufacturers, so monopoly was more common for these kinds of medicines (Cheng and Li, 2016; He et al., 2018). And when no ceiling price was set, medicine prices may increase considerably. Hence, we assumed that NPMPP would increase prices for paediatric, gynaecological, and emergency medicines.

The medicine price directly affected healthcare cost (Wang and Long, 2010). Currently, the continuous increasing of healthcare expenditure was one of the biggest healthcare issues in China (Yan et al., 2019). The per capita expenditure on healthcare for urban residents rose from 1,136 RMB ($169.14, $1 = 6.7162RMB on March 23, 2019) in 2013 to 1,777 RMB ($264.58) in 2017, with a growth rate of 56% during these 5 years in China (The State Council, 2018). Among the total healthcare expenditure, the pharmaceutical cost was one essential constituent. Even in 2017, the zero markup drug policy was implemented in all the hospitals (previously, hospitals were allowed to earn a 15% markup on the sale of pharmaceutical products); for inpatient, the pharmaceutical expenditure still took account for more than 30% of the total cost; for outpatient, the proportion was 42.7% (NHFPC, 2018). The problem of huge pharmaceutical cost was serious, and rapidly rising medicine price may make it worse.

To the best of our knowledge, the effect of this NPMPP on medicine price has not been characterised. In the context of increasing pharmaceutical expenditure in China, identifying and understanding the unintended consequences of this NPMPP on medicine price were highly relevant. Thus, the aim of this study was to assess the impacts of the NPMPP on medicine price.

## Methods

### Study Design

The NPMPP was introduced by China’s NHFPC in January 2015 (SNHFPC, 2015). After the announcement of this policy, they provided a medicine list recommending for using this procurement scheme in September of the same year. This medicine list contained only paediatric, gynaecological, and emergency medicines. After checking all the provincial policies, we found that up to now 23 provinces have implemented this NPMPP. However, the implementation time and medicine lists varied among different provinces. To ensure the veracity of assessment of this NPMPP, we focused on one single province, Shaanxi Province, by monitoring contextual factors that might influence our interests.

Shaanxi Province was chosen for study because of the following reasons. First, Shaanxi Province was one of the earliest provinces implementing the NPMPP, which guaranteed enough data for evaluation. Following the NHFPC’s version of NPMPP, the Shaanxi Health and Family Planning Commission issued a corresponding document on the NPMPP in August 2015 and announced the first batch of medicines that would be applied to this policy since April 2016 (SNHFPC, 2015). Second, Shaanxi was broadly representative of the typical health and health system status of the 12 western provinces of China. It has a population of 38.35 million and 11 areas in its jurisdiction, ranked 14th for gross domestic product per capita in Mainland China (31 provinces in total in the Mainland) in 2017 (The State Council, 2018). In Shaanxi Province, there were 1,150 hospitals, 33,810 basic medical and health institutions, and 800 specialty public health agencies (**Table 1**). Third and most important, the Shaanxi Provincial government was able and willing to collaborate in this study.

**Table 1:** Comparative characteristics of Shaanxi Province and the whole country, China, 2017.

Characteristic	Shaanxi Province	China
Population	383,500,000	13,900,800,000
Hospitals	1,150	31,056
Basic medical and health institutions	33,810	933,024
Specialty public health agency	800	19,896

Source: China Statistical Yearbook of 2018 and Shaanxi Statistical Yearbook of 2018 (The State Council, 2018; Shaanxi’s Statistical Bureau, 2018).

Similar to NHFPC, the first batch of medicines in Shaanxi still contained only paediatric, gynaecological, and emergency medicines. Later, in 2017 some common low-priced medicines and basic infusions were added in the new procurement processing, and ceiling prices were set for these medicines. In this article, we tried to assess the impact of NPMPP on the prices of paediatric, gynaecological, and emergency medicines.

### Medicine Selection

One medicine was selected for study only when it was both in the medicine list recommended by the China NHFPC and the first batch of medicines announced by the Shaanxi Health and Family Planning Commission. Finally, 95 medicines (same medicine name, different dosage forms or strengths were considered as different medicines) were selected, including 55 emergency medicines, 14 gynaecological medicines, and 26 paediatric medicines.

### Data Collection and Management

Data were gathered from the Shaanxi Public Health Administration Department, which was responsible for centralised transactions in pharmaceutical and medical products procurement in public hospitals in Shaanxi. We extracted monthly purchasing information (procurement price and quantity) of all the public health institutions in Shaanxi Province for the 95 medicines, from January 2015 to December 2017. It would be taken as missing data for 1 medicine if no hospital purchased this medicine in a certain month.

Among the 95 medicines, we retained 35 medicines (including 22 emergency medicines, 7 gynaecological medicines, and 6 paediatric medicines), and 59 medicines were excluded because of missing data. The exclusion criterions were as follows: 1) if no data were available for more than three consecutive months for 1 medicine, then this medicine would not be included in the analysis; 2) if no data were available for more than 6 months for 1 medicine in total, then this medicine would be excluded.

For the final 35 medicines (**Table 2**), when 1 medicine had no purchasing record in a certain month, we used the purchase price of the previous month to represent the price and set quantity as zero for the current month. For better comparison, all the price data were adjusted according to the consumer price index.

**Table 2:** The information of all the 35 medicines.

Category	Name	Dosage forms	Strengths
**Emergency medicines**	Norepinephrine	Injection	1 ml: 2 mg
	Metaraminol bitartrate	Injection	1 ml: 10 mg
	Phentolamine mesylate	Injection	1 ml: 10 mg
	Sodium nitroprusside	Sterile powder for injection	50 mg
	Nitroglycerine	Injection	1 ml: 5 mg
	Tranexamic acid	Injection	5 ml: 0.25 g
	Suxamethonium chloride	Injection	2 ml: 100 mg
	Vecuronium bromide	Sterile powder for injection	4 mg
	Haloperidol	Injection	1 ml: 5 mg
	Magnesium sulphate	Injection	10 ml: 2.5 g
	Neostigmine Methylsulphate	Injection	2 ml: 1 mg
	Flumazenil Flumazenil Flumazenil	Injection Injection Injection	2 ml: 0.2 mg
			5 ml: 0.5 mg
			10 ml: 1 mg
	Naloxone hydrochloride Naloxone hydrochloride	Injection	1 ml: 0.4 mg
		Injection	2 ml: 2 mg
	Methylthioninium Chloride	Injection	2 ml: 20 mg
	Carbonis medicinalis	Tablets	0.3 g
	Tetanus antitoxin	Injection	1,500 IU
	Potassium chloride	Injection	10 ml: 1.5 g
	Sodium bicarbonate Sodium bicarbonate	Injection Injection	10 ml: 0.5 g
			250 ml: 12.5 g
**Gynaecological medicines**	Metronidazole	Tablets	0.2 g
	Clotrimazole	Tablets	0.5 g
	Chorionic gonadotrophin	Injection	1,000 IU
	Norethisterone	Tablets	0.625 g
	Mifepristone	Tablets	25 mg
	Magnesium sulphate	Injection	10 ml: 2.5 g
	Misoprostol	Tablets	0.2 g
**Paediatric medicines**	Amoxicillin	Granules	0.125 g
	Cefixime Cefixime	Suspension	50 mg
		Granules	50 mg
	Ibuprofen Ibuprofen	Suspension Suspension	60 ml: 1.2 g
			100 ml: 2 g
	Cyclosporine	Capsules	25 mg

### Outcome Measurements

To analyse the changes in drug price, we adopted purchasing price growth rate to describe the speed of price change. The price growth rate of drug *j* in month *i* was calculated according to the following equation:

$$r_{ij}\;=(p_{ij}-p_{0j})/p_{0j}$$

where *p*_0_ *_j_* means the average purchasing price of drug *j* in the first month (January 2015) for all the public healthcare institutions in Shaanxi; *p* *_ij_* means the average purchasing price of drug *j* in the *i*th month. The monthly average price growth rate for each category was defined as MPRe (for emergency medicines), MPRg (for gynaecological medicines), and MPRp (for paediatric medicines). And the MPRe in month *i* was calculated as follows:

MPRei=∑j∈Jrij22(Jisthesetofthe22emergencymedicines)

Similarly, MPRg*_i_* and MPRp*_i_* were calculated.

### Statistical Analysis

Interrupted time series analysis was considered to be the strongest, quasi-experimental design to evaluate the longitudinal effects of time-delimited interventions (Cook and Campbell, 1979; Rashidian et al., 2013). Segmented regression analysis of interrupted time series data can statistically assess how much an intervention changed an outcome of interest, transiently or in the long term (Wagner et al., 2002).

We carried an interrupted time series analysis of the price growth rate on each category of medicines. For each category of medicines, two segments with one interruption point were constructed. As the NPMPP was implemented in April 2016, the first segment was February 2015 (January 2015 was considered as base month; there were no MPRe/MPRg/MPRp values in this month) to March 2016 (14 months before intervention). To avoid incorrect specification of intervention effects, we excluded April 2016 from the analysis as it was a month lag in the effect of the intervention. Hence, the second segment was from May 2016 to December 2017 (20 months after the intervention). We assumed that there could be an immediate increase in the price increasing rate in May 2016 for the three categories of medicines, and the monthly trend in the price increasing rate after the intervention could differ from that before the intervention. We also assumed that the trend in the increasing price rate after intervention would differ among different kinds of medicines.

We tested for serial correlation by assuming a first-order autoregressive correlation structure. Breusch–Pagan statistic was used to check for heteroscedasticity in the residuals, and when heteroscedasticity existed, robust regression was adopted to correct the heteroscedasticity. We used Stata SE 12.0 (Stata Corporation, College Station, TX, USA), for estimation.

## Results

**Figure 1:**
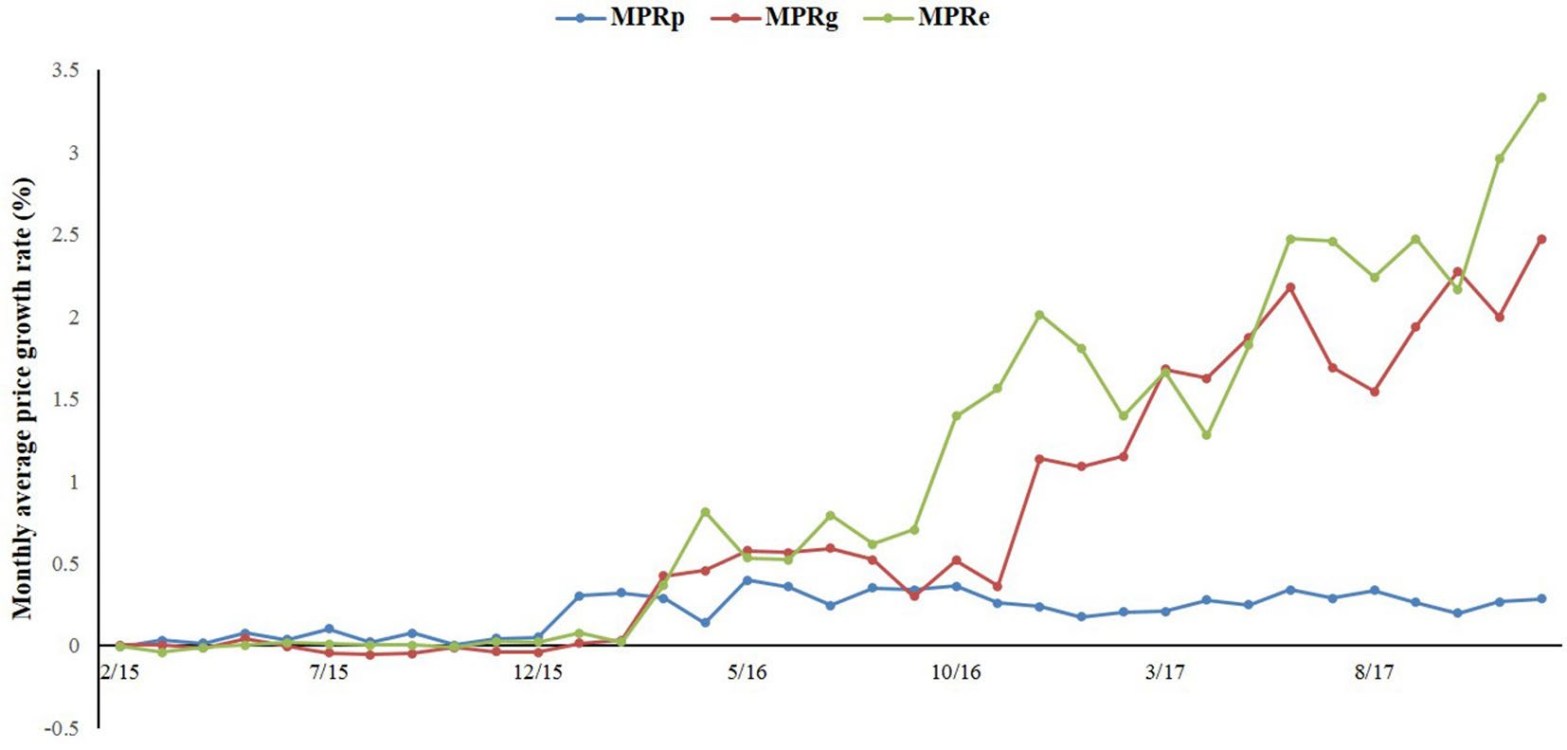
The trend in the monthly average price growth rate of paediatric, gynaecological and emergency medicines in Shaanxi Province from 2015 to 2017.

As **Figure 1** shows, the overall prices of paediatric, gynaecological, and emergency medicines were increasing during the period of 2015 to 2017. Among the three categories of medicines, the prices of gynaecological and emergency medicines increased dramatically after the intervention, whereas the price of paediatric medicines was relatively stable.

### Monthly Average Price Growth Rate of Emergency Medicines

**Figure 2:**
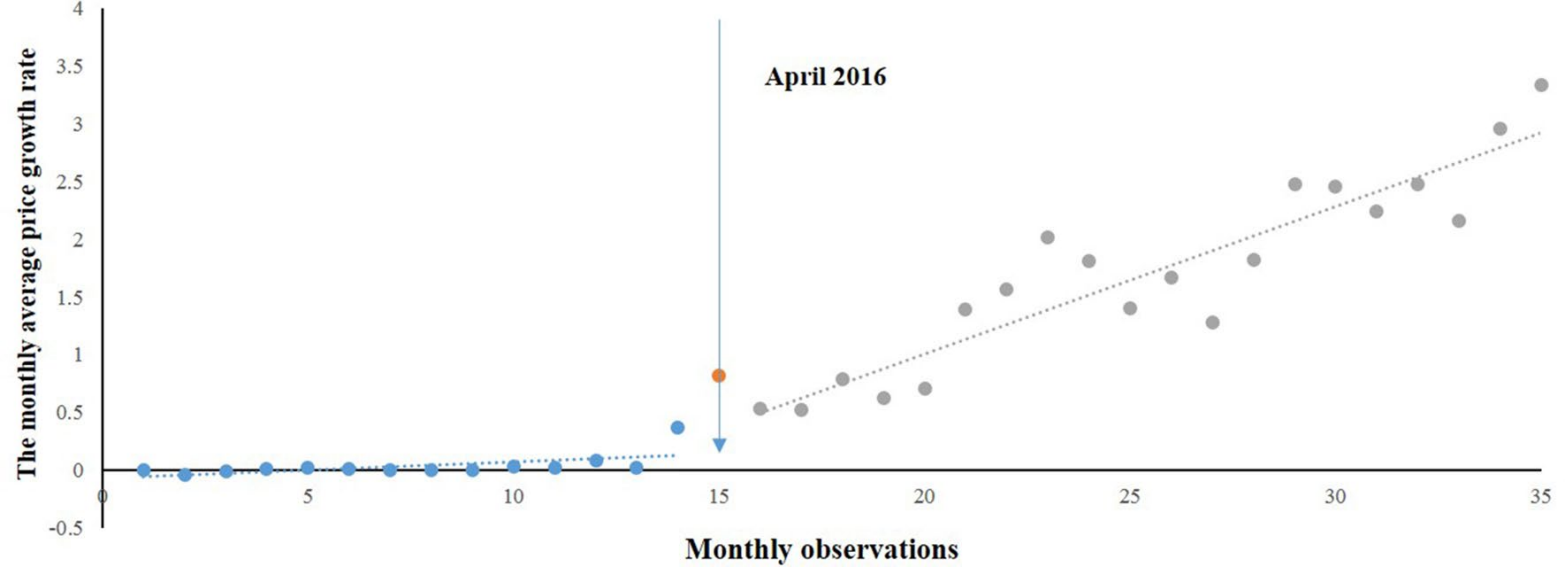
Segmented regression model showing MPRe from 2015 to 2017.

**Figure 2** shows the time series of MPRe from 2015 to 2017. Before the implementation of NPMPP, there was a slight increasing trend in the MPRe. After the intervention, a little change in level was noted, and change can also be observed in the trend. We developed a regression model for MPRe:

$$\text{MPRe}=-0.069+0.014^{*}T+0.243^{*}D+0.114^{*}P$$

where *T* was a preintervention slope, *P* was the change in slope, and *D* was the change in the intercept.

As **Table 1** shows, we found an increasing rate (0.014) in the MPRe from month to month before the intervention (*P* = 0.088). After the intervention, significant increases in the regression slope (coefficient = 0.114, *P* < 0.001) and in the level were noted (coefficient = 0.243, *P* = 0.096).

### Monthly Average Price Growth Rate of Gynaecological Medicines

**Figure 3:**
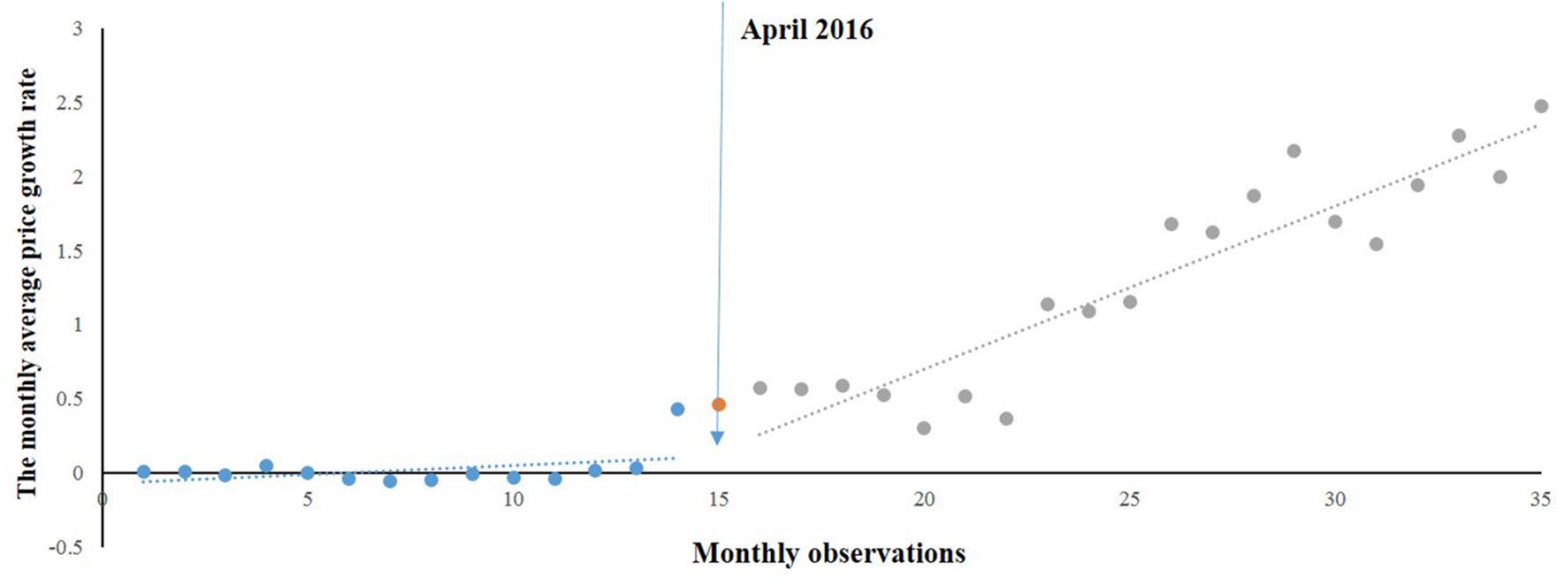
Segmented regression model showing MPRg from 2015 to 2017.

According to the time series of monthly average price growth rate of gynaecological medicines (**Figure 3**), changes in the level and trend were similar to those of emergency medicines. A regression model was developed:

MPRg^=−0.095+0.021∗T+0.026∗D+0.078∗P

where MPRg^^^ was the MPRg after correcting for autocorrelation (MPRg*_t_* − *q* * MPRg*_t_*_−1_), and *q* was the autocorrelation parameter and equaled 0.375.

Likewise, a positive but no significant rise in **Table 3** was observed in the MPRg from month to month before the intervention. After the intervention, a significant increase (0.078) in the regression slope (*P* < 0.05) was noted. The level also showed an increase (0.026) but not statistically significant (*P* = 0.9).

**Table 3:** Estimated coefficients of segmented regression models for the MPRe, MPRg, and MPRp before and after the NPMPP, Shaanxi Province, February 2015 to December 2017.

Parameter	MPRe		MPRg		MPRp	
	Value (RSE)	*P*	Value	*P*	Value	*P*
Intercept	–0.069	0.112	–0.095	0.694	–0.061	0.428
Preintervention slope	0.014	0.088*	0.021	0.412	0.019	0.025**
Change in intercept	0.243	0.096*	0.026	0.900	0.100	0.103
Change in slope	0.114	0.000***	0.078	0.013**	–0.024	0.013**

RSE, robust standard error. Two-tailed P value: *P < 0.10, **P < 0.05, ***P < 0.01.

### Monthly Average Price Growth Rate of Paediatric Medicines

**Figure 4:**
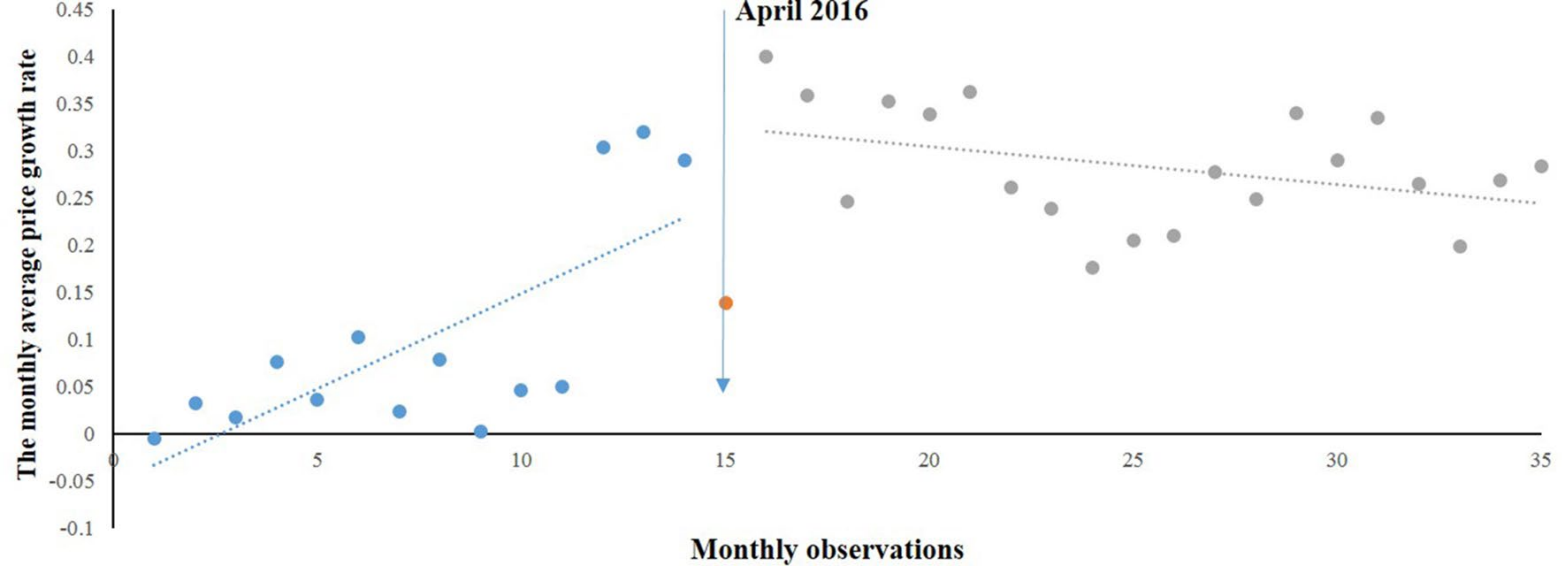
Segmented regression model showing MPRp from 2015 to 2017.

As shown in **Figure 4**, the trend of MPRp was stable during the whole period. A regression model was also generated:

$$\text{MPRp\textasciicircum{} = -0.061 + 0}{\text{.019}}^{*}T+0.100^{*}D-0.024^{*}P$$

where MPRp^^^ was the MPRp after correcting for autocorrelation (MPR*p* *_t_* − *q* * MPR*p* *_t_*_−1_), and *q* was the autocorrelation parameter and equaled 0.428.

The trend of MPRp was different from MPRe and MPRg. Before the intervention, a significant increase (0.019) was found in the average price growth rate of paediatric medicines (*P* = 0.025). However, a significant decrease (−0.024) was noted in the regression slope (*P* < 0.05) after the intervention. An increase (0.100) but not statistically significant (*P* = 0.103) in the level was found.

## Discussion

In this study, we identified the effects of the NPMPP, which aimed to address this drug shortage problem, on the price of emergency, gynaecological, and paediatric medicines. Most of the previous studies related to NPMPP were about the implementation mode of this policy in different provinces (Chen, 2017; Fang et al., 2015). To the best of our knowledge, this was the first quantitative study to investigate the effects of the NPMPP on medicine price in China. An interrupted time series analysis showed the NPMPP in Shaanxi Province resulted in an acceleration of the price growth rate of emergency medicines and gynaecological medicines, but a slight deceleration of the price growth rate of paediatric medicines. The changes in price growth rate varied; however, the medicines prices of the three categories were still higher compared with the prices before the intervention.

Before the introduction of NPMPP, the monthly average price growth rates of the three categories of medicines revealed a trend of slow increase. The intervention accelerated the monthly average price growth rates of emergency medicines and gynaecological medicines significantly. The relative increased rate of MPRe (0.114/0.014) was bigger than that of MPRg (0.078/0.021). This suggested that the impact of on MPRe was greater than that on MPRg. However, the NPMPP decreased the monthly average price growth rate of paediatric medicines. We noted two possible explanations for this result. First, the median of the number of manufacturers for emergency medicines and gynaecological medicines was 4, which was fewer than that for paediatric medicines (eight suppliers). Therefore, the monopoly was more common for emergency and gynaecological medicines. While for paediatric medicines, the NPMPP enlarged the number of suppliers, intensified the competition between manufacturers, and finally resulted in a decrease in the price growth rate. Second, the prices of active ingredients for many emergency medicines increased greatly during last 2 years (Baidu Research, 2019; Financial Network, 2019; Quanzhou Network 2019). For example, the price of inosine (an active ingredient for leukopenia, various heart diseases, chronic hepatitis, cirrhosis, etc.) rose from 92 to 95 RMB/kg in early 2015 to 600 RMB/kg in July 2018 (Zhan, 2018), and the price of raw materials of nitroglycerin injection rose sharply, almost tripled from 2015 to 2018 (Sina Finance, 2019).

Although we observed a decreasing trend in the MPRp, the prices of all the 35 medicines were still increasing after the NPMPP. This result was different compared with a previous study. Based on the annual tender prices of medicines in 31 provinces, Shang et al. (2019) found that there was an instant increase for the overall price of these medicines (including emergency, gynaecological, and paediatric medicines; common low-priced medicines and basic infusions, recommended using NPMPP by China’s NHFPC) in 2015, but a stable decrease from 2016 to 2017. This difference could be attributed to the use of different methods, as well as different medicine samples and different study areas. Especially in China, there was a great disparity of medicine prices among different provinces, while Shaanxi was ranked fourth for high drug price (Pan and Wang, 2019). Therefore, using the average price data of 31 provinces would generate different results, comparing with our research, which included price information only of Shaanxi Province.

The study design had two advantages. First, no other related policies were implemented during 2015 to 2017, which guaranteed that we can perform a natural experiment and use robust research method. Second, we controlled for possible confounding by using the price information for all the public healthcare institutions in Shaanxi Province and taking the monthly average price growth rate for each category as our outcome indicator.

Limitations of this study included that the study was conducted in only one province, whereas ideally it should have been conducted in a nationally representative sample. However, the NPMPP was carried out on a province level, which means that the implementation time and drug catalogue were different in different provinces. Hence, it was difficult to extend our research to the whole country. The second limitation was the lack of a control group. Although it was not necessary to build a control group to establish a causal relationship between an intervention and an outcome, the control group may help better understand the effects of the intervention. On the other hand, the choice of medicines in the control group would be subjective, which may lead to bias.

Our study had important policy implications. The NPMPP needs to be modified to restrain the upward trending in prices of emergency and gynaecological medicines. Generally, no ceiling price needs to be cautiously considered, especially for medicines with a small number of manufacturers and for countries with no strong antitrust regulation.

## Conclusion

We investigated the effects of an NPMPP on medicine price in Shaanxi Province, western China. By interrupted time series analysis, we identified a statistically significant increase in the price growth rate of emergency medicines and gynaecological medicines, but a statistically significant decrease in the price growth rate of paediatrics, following the introduction of NPMPP. The impact of NPMPP on emergency medicines was greater than that on gynaecological medicines. Other effective policies were needed to inhibit the price growth rates of emergency and gynaecological medicines.

## Data Availability

The data analyzed in this study was obtained from Shaanxi Health and Family Planning Commission. Requests to access these datasets should be directed to Caijun Yang, yangcj@xjtu.edu.cn.

## Author Contributions

Conceived and designed the experiments: CY, SH. Performed the experiments: SH, CC. Analysed the data: SH, CC, CY, SY, FX. Wrote the paper: SH. Critical revision of the manuscript: SH, CC, CY, SY, FX, LS. Approval of the final version of the manuscript: SH, CC, CY, SY, FX, LS, YF.

## Funding

This work is supported by the National Natural Science Foundation of China (7150319) and “the Fundamental Research Funds for the Central Universities.”

## Conflict of Interest Statement

The authors declare that the research was conducted in the absence of any commercial or financial relationships that could be construed as a potential conflict of interest.
